# Supplementary material for: Heparin-based hydrogel scaffolding alters the transcriptomic profile and increases the chemoresistance of MDA-MB-231 triple-negative breast cancer cells
Source: Biomater Sci. 2020 Feb 13;8(10):2786–96. doi: 10.1039/c9bm01481k (PMC7497406; doi:10.1039/c9bm01481k)
Supplement: Supplementary file 2 [file BM-008-C9BM01481K-s002.zip › Supplementary File 4/HP-BhydrogelvControl/Pathways/my_analysis.Gsea.1545201079907/HALLMARK_APICAL_SURFACE.html]

Details for gene set HALLMARK\_APICAL\_SURFACE[GSEA]

|  || Dataset | expr.class.cls#HYD\_versus\_CONTROL.class.cls#HYD\_versus\_CONTROL\_repos |
| Phenotype | class.cls#HYD\_versus\_CONTROL\_repos |
| Upregulated in class | HYD |
| GeneSet | HALLMARK\_APICAL\_SURFACE |
| Enrichment Score (ES) | 0.3583435 |
| Normalized Enrichment Score (NES) | 1.0794836 |
| Nominal p-value | 0.3465649 |
| FDR q-value | 0.78297657 |
| FWER p-Value | 1.0 |
Table: GSEA Results Summary

  

Fig 1: Enrichment plot: HALLMARK\_APICAL\_SURFACE      
 Profile of the Running ES Score & Positions of GeneSet Members on the Rank Ordered List

  

| PROBE | DESCRIPTION (from dataset) | GENE SYMBOL | GENE\_TITLE | RANK IN GENE LIST | RANK METRIC SCORE | RUNNING ES | CORE ENRICHMENT || 1 | SLC34A3 | na |  |  | 52 | 3.643 | 0.1401 | Yes |
| 2 | MDGA1 | na |  |  | 410 | 2.460 | 0.2179 | Yes |
| 3 | CROCC | na |  |  | 1135 | 1.774 | 0.2497 | Yes |
| 4 | SCUBE1 | na |  |  | 1225 | 1.723 | 0.3125 | Yes |
| 5 | EPHB4 | na |  |  | 1524 | 1.565 | 0.3583 | Yes |
| 6 | PCSK9 | na |  |  | 3627 | 0.886 | 0.2834 | No |
| 7 | AFAP1L2 | na |  |  | 3697 | 0.871 | 0.3140 | No |
| 8 | SHROOM2 | na |  |  | 4267 | 0.758 | 0.3140 | No |
| 9 | CD160 | na |  |  | 5188 | 0.602 | 0.2896 | No |
| 10 | GATA3 | na |  |  | 5553 | 0.545 | 0.2920 | No |
| 11 | ATP6V0A4 | na |  |  | 6256 | 0.450 | 0.2730 | No |
| 12 | NCOA6 | na |  |  | 7082 | 0.350 | 0.2437 | No |
| 13 | TMEM8B | na |  |  | 7548 | 0.300 | 0.2312 | No |
| 14 | AKAP7 | na |  |  | 7604 | 0.292 | 0.2398 | No |
| 15 | CX3CL1 | na |  |  | 8580 | 0.196 | 0.1966 | No |
| 16 | FLOT2 | na |  |  | 10101 | 0.029 | 0.1184 | No |
| 17 | EFNA5 | na |  |  | 10800 | -0.037 | 0.0835 | No |
| 18 | PLAUR | na |  |  | 11509 | -0.112 | 0.0509 | No |
| 19 | BRCA1 | na |  |  | 12249 | -0.206 | 0.0205 | No |
| 20 | ATP8B1 | na |  |  | 13410 | -0.339 | -0.0268 | No |
| 21 | B4GALT1 | na |  |  | 13415 | -0.340 | -0.0137 | No |
| 22 | LYN | na |  |  | 13668 | -0.380 | -0.0119 | No |
| 23 | APP | na |  |  | 13765 | -0.397 | -0.0014 | No |
| 24 | HSPB1 | na |  |  | 15951 | -0.733 | -0.0866 | No |
| 25 | ADIPOR2 | na |  |  | 16920 | -0.962 | -0.0994 | No |
| 26 | GSTM3 | na |  |  | 17268 | -1.074 | -0.0754 | No |
| 27 | DCBLD2 | na |  |  | 17598 | -1.196 | -0.0457 | No |
| 28 | ADAM10 | na |  |  | 18289 | -1.557 | -0.0206 | No |
| 29 | SRPX | na |  |  | 18537 | -1.735 | 0.0345 | No |
Table: GSEA details [plain text format]

  

Fig 2: HALLMARK\_APICAL\_SURFACE      
 Blue-Pink O' Gram in the Space of the Analyzed GeneSet

  

Fig 3: HALLMARK\_APICAL\_SURFACE: Random ES distribution      
 Gene set null distribution of ES for **HALLMARK\_APICAL\_SURFACE**
